# Fungal disease in AIDS patients in intensive care

**DOI:** 10.1186/cc12941

**Published:** 2013-11-05

**Authors:** Edwiges Santos, Andre Japiassu, Marcia Lazera, Fernando Bozza

**Affiliations:** 1 Instituto Evandro Chagas, Fiocruz, Brazil

## Background

Information about the prevalence of fungal diseases in critically ill AIDS patients is sparse. Our goal is to describe the prevalence of fungal diseases in this population, when they are admitted to the ICU.

## Materials and methods

Prospective, observational study. Blood and urine samples were collected from 65 AIDS patients at a specialized ICU in infectious diseases, from August 2011 to June 2013. When indicated by clinical suspicion, samples of respiratory, bone marrow and/or tissue were collected. Cultures, cytopathology and serologic tests were performed to evaluate fungal colonization or infection. Clinical data were collected from medical records. Values are expressed as the median and percentage.

## Results

**Figure 1:**
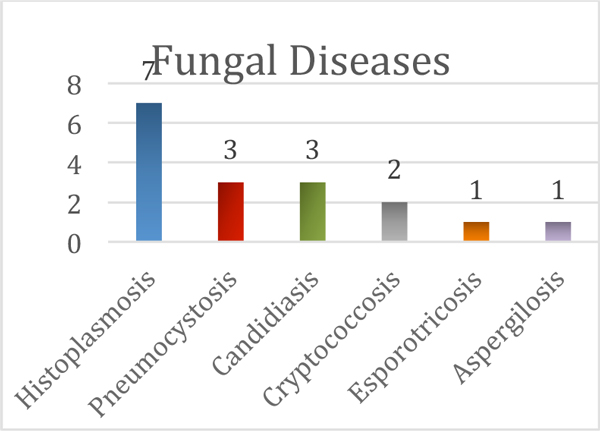
**Fungal diseases**.

Table 1 presents general characteristics of the HIV/AIDS patients. Patients with fungal disease did not differ from patients without fungal infection: age 35 versus 38 years (*P *= 0.43), male gender 76% versus 70% (*P *= 0.29); nadir CD4 cell count 27 versus 57 cell/mm^3 ^(*P *= 0.15). Most patients were exposed to HAART previously, while there were 47% naïve patients in the fungal group versus 31% in the no fungal group. The ICU mortality of patients without fungal disease was 31% versus 64.7% with fungal disease (*P *= 0.02); hospital mortality was not different between groups (52% vs. 64.7%, *P *= 0.4). Figure 1 presents 17 diagnoses of disseminated fungal diseases (prevalence 26%). All histoplasmosis diagnoses were made from marrow bone culture (11%). Disseminated cryptococcosis was diagnosed from serum serologic latex, direct examination and positive culture in LCR. Three patients (4.6%) were diagnosed with candidiasis in blood cultures. Pnemunocystosis was diagnosed from immunofluorescence and Grogot positive in sputum. One patient had disseminated esporotricosis with positive cultures in LCR, blood, tissue, urine and sputum. The only case of aspergilosis is a previous tuberculosis-treated patient that developed a disseminated disease (galactomanana-positive) from a fungal ball.

**Table 1:** Population characteristics

	No fungal (*n *= 48)	Fungal (*n *= 17)	*P *value
Age (years)	38 (31 to 43)	35 (33 to 46)	0.43
Gender (male)	34 (71%)	13 (76%)	0.29
CD4^+ ^lymphocyte count (cell/mm^3^)	69 (32 to 204)	28 (14 to 115)	0.15
Nadir CD4^+ ^(cell/mm^3^)	57 (27 to 153)	27 (14 to 122)	0.40
Time since HIV diagnosis (months)	31 (1 to 123)	13 (1 to 77)	0.53
HAART näive	15 (31%)	8 (47%)	0.56
Mortality	15 (31%)	11 (64.7%)	0.02

## Conclusions

One in four HIV/AIDS critically ill patients presents with fungal disease when they are admitted to the ICU. Surveillance of fungal pathogens shall be necessary in the first screening of medical HIV/AIDS patients in the ICU.

